# Evaluating the role of polysaccharide extracted from *Pleurotus columbinus* on cisplatin-induced oxidative renal injury

**DOI:** 10.1038/s41598-022-27081-2

**Published:** 2023-01-16

**Authors:** Sahar S. Mohamed, Ghada S. Ibrahim, Mona A. M. Ghoneim, Amal I. Hassan

**Affiliations:** 1 Microbial Biotechnology Department, Biotechnology Research Institute, National Research Centre, Dokki, Cairo, Egypt; 2 Department of Radioisotopes, Nuclear Research Centre, Egyptian Atomic Energy Authority, Cairo, Egypt

**Keywords:** Biological techniques, Biotechnology, Health care

## Abstract

This research aimed to examine the antioxidant polysaccharide activity (PsPc-3) derived from *Pleurotus columbinus* (*P. columbinus*) on oxidative renal injury (ORI) induced by cisplatin (CP). The principal components of crude polysaccharide were assessed. We studied the preventive impact of polysaccharide on cisplatin-induced renal damage in this study. For 21 days, we employed the CP-induced ORI rat model and divided the rats into four groups: control, CP alone, polysaccharide post CP (100 mg/kg) orally, and CP + polysaccharide (pre and post). The chemical characterization of the polysaccharide fraction PsPc-3 stated that protein was not present. PsPc-3 contained 7.2% uronic acid as assessed as 0% sulfate. PsPc-3 hydrolysate structured of Galacturonic:Glucose:Xylose and their molar proportions were 1:4:5, respectively. The average molecular weight (Mw) and molecular mass (Mn) per molecule of PsPc-3 were 5.49 × 10^4^ g/mol and Mn of 4.95 × 10^4^ g/mol respectively. DPPH radical scavenging activity was demonstrated by the polysaccharide of 65.21–95.51% at 10 mg/ml with IC50 less than 10 mg/ml. CP increased serum urea to 92.0 mg/dl and creatinine up to 1.0 mg/dl, with a concurrent decrease in the levels of total protein to 4.0 mg/dl. Besides, Also, CP-induced ORI raised levels of malondialdehyde (MDA), alkaline phosphatase (ALP), and renal hormones (renin and aldosterone), with a decline in antioxidants compared to control rats. In addition, in the presence of CP, interleukin-6 (IL-6) and tumour necrosis factor-alpha (TNF-alpha) levels increased. PsPc-3 decreased these changes dramatically. PsPc-3 improves pathological renal damage caused by CP and decreases tubular apoptosis measured by DNA ladder formation and cleaved caspase- 3. These findings showed that PsPc-3 isolated from *P. columbinus* protects and inhibits tubular apoptosis in cisplatin-induced ORI. Furthermore, PsPc-3 has no influence on the anticancer efficacy of CP in rats. Thus, PsPc-3 derived from *P. columbinus* might provide a novel therapy method for cisplatin-induced nephrotoxicity.

## Introduction

Cisplatin (dichlorodiamino platinum) is one of the chemotherapy drugs, which is used for the treatment of a variety of solid malignant tumors encompassing the testis, lung, head, neck, and bladder cancers^1^. Cisplatin usage is also restricted by numerous significant adverse outcomes such as suppression of bone marrow, neuropathy, and nephrotoxicity^2^. Improvement of the awareness of the pathogenesis of acute renal failure (ARF) caused by cisplatin is crucial if cisplatin-based treatment is to avoid ARF and enhance the survival of cancer patients. Polysaccharides are the most abundant group of natural, non-toxic biopolymers, isolated from various flora and fauna have been found to participate in many biological processes^3^. Mushrooms have recently gained popularity not only as a protein-rich health vegetable (food) but also as a source of physiologically active therapeutic value chemicals. Polysaccharides, antioxidants, and lectins are extremely important new pharmaceutical items for medicinal mushrooms^4^.

Polysaccharides, which are generated by fungus, have a variety of biological capabilities, including antioxidant, antitumor, antibacterial, anti-inflammatory, immunomodulatory, anti-hyperglycemic, and anti-hypercholesterolemic effects^5^. As potential protective agents, the antioxidants present in mushrooms are of great importance in helping the human body mitigate oxidative damage without intervention^6^. Moreover, some studies suggest that the possible results of their exploitation as novel therapies in some diseases were the polysaccharides derived from *Agrocybe cylindracea*^7^, *Pleurotus eryngii*^8^ and Flamolina phylotypes from SMC^9^. As well as polysaccharides in the fungus PSP improve white blood cell effectiveness present in the peripheral vessels, then induces those cells to secrete interleukin-6, and stimulate the work of specialized white blood cells. Consequently, it increases the alpha- and gamma-type interferons by 2–4 times. *Pleurotus *spp. is pharmaceutical mushrooms as antiviral, antitumor, antibiotic, antibacterial, hypercholesterolemia, and immunomodulating agent^10^.

The polysaccharides extracted from *Pleurotus* have shown therapeutic effects in cancer cells by preventing these cells from proliferating such as the cell of colon cancer Caco-2, hepatoma cell HepG2, cell of colon cancer type HT-29, and human fetal kidney cells 293T^11–14^. *Pleurotus* polysaccharides also display a defensive effect in a linoleic-acid modeling method against auto-oxidation^15^. Besides, powerful free radicals scavenging and substantial activities against bacteria as well as polysaccharide's anti-inflammatory properties taken from different *Pleurotus *spp. in publications^16^.

Pleurotus fruiting tissues have a greater antioxidant content than comparable commercial mushrooms^17^. This behavior was caused mostly by the existence of *P. ostreatus* isolated Pleuran, a polysaccharide (-glucan), which has been shown to benefit rats' colons with precancerous lesions^18^. *P. ostreatus* increases the activity of significant enzymes of antioxidation (such as catalase, peroxidase, and superoxide dismutase), minimizing human oxidative destruction^19^. Because of their increased free radical scavenging properties, oyster mushrooms are increasingly commonly employed as components in dietary supplements in the hopes of preserving health and avoiding disease^20^ due to their higher activities that scavenge free radicals^21^.

Towards the biological effect of polysaccharide from *Pleurotus columbinus* on cisplatin-induced oxidative renal injuries, this study was planned to investigate these findings.

## Results

### Isolation and chemical structure of PsPc-3 from *Pleurotus columbinus*

**Figure 1:**
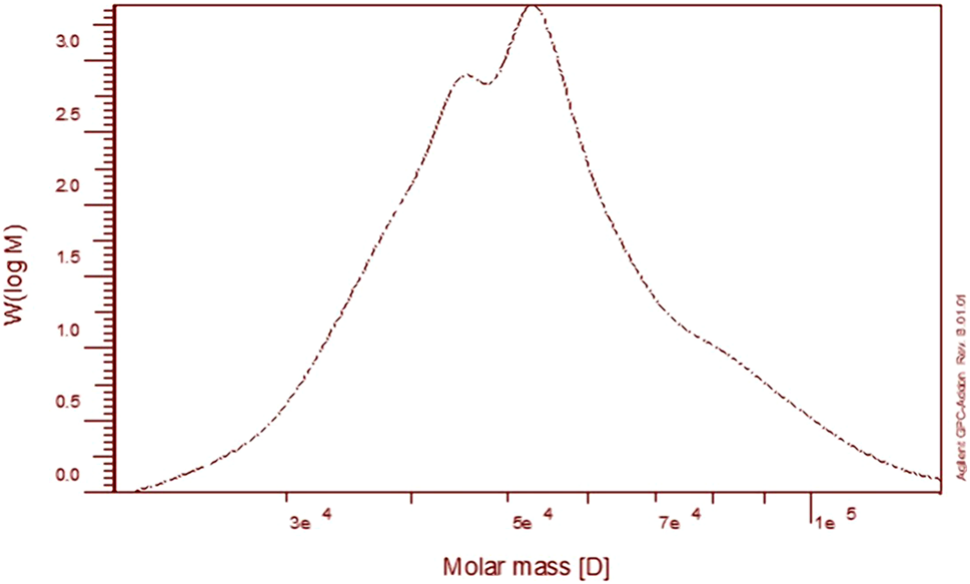
The molecular weight distribution of PsPc-3 from *Pleurotus columbinus.*

*Pleurotus columbinus* PS output was 5.55 g/100 g dry weight. After ethanol fractionation, crude PS precipitated, the primary fraction called PSPc-3 was achieved. The PSPc-3 has been gathered for further structural assessment. It emerged as a white-yellow powder, with the Bradford test having an adverse response. The data that the UV spectra did not detect absorbance between 260 and 280 nm stated the particular protein was not present. PSPc-3 contained 7.2 % uronic acid as assessed by colorimetric m-hydroxydiphenyl and 0 % sulfate. HPLC determined the structure of monosaccharides of PSPc-3 hydrolysate, in which Galacturonic:Glucose:Xylose was recognized in respect to their molar ratios in the hydrolysate were 1: 4: 5, respectively. Smiderle et al.^22^ and Silveira et al.^23^ were found to have a marked ant nociceptive impact when tested in mice, showing that mushroom heteropolysaccharides may also have therapeutic characteristics such as anti-inflammatory activity. By using chromatography of gel permeation, the PSPc-3 Mw, Mn, and polydispersity index (PI) were assessed. The PSPc-3 was widely dispersed (PI) 1.11 in the GPC chromatogram and had an average Mw 5.49 × 10^4^ g/mol and Mn 4.95 × 10^4^ g/mol (Fig. 1).

### Fourier transform infrared analysis (FT-IR)

**Figure 2:**
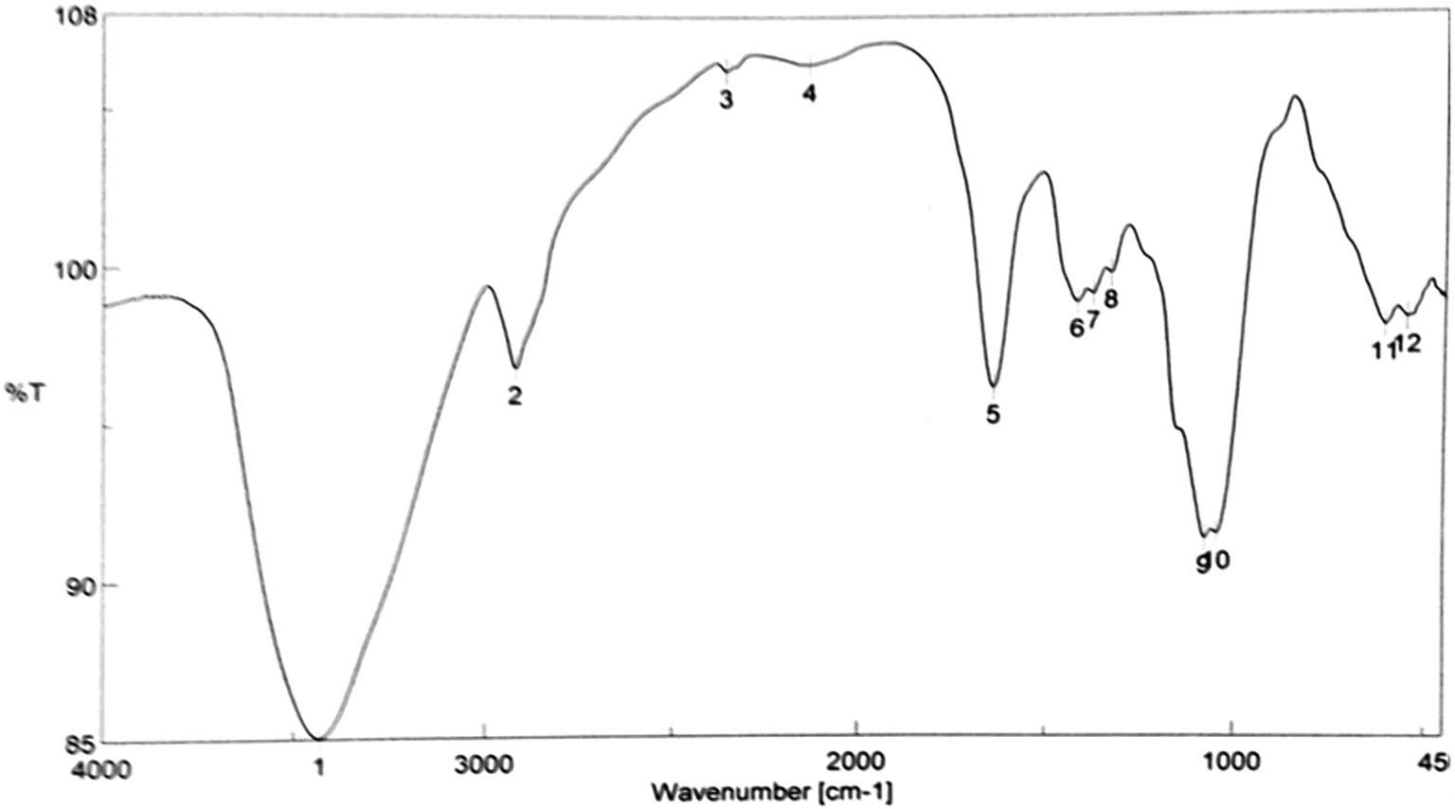
FT-IR spectrum of PsPc-3 from *Pleurotus columbinus.*

FT polysaccharide-IR is an efficient strategy that is now utilized to identify functional groups and describe covalent bonding. The FTIR spectra of the PsPc-3 as indicated in Fig. 2 exhibited a wide hydroxyl (O–H) group stretching vibration at 3434.6 cm^−1^, indicating the existence of the OH axial deformation that matched the intermolecular and intermolecular hydrogen bond^24,25^. It is well known that the hydroxyl groups of carbohydrates are what give EPS its characteristics and solubility^26^. The secondary and primary (CH_2_) bands at 2924.51 cm^−1^ have soft bands that could be explained by the C–H stretching of methyl or methylene groups in hexoses like glucose or deoxyhexoses like rhamnose^27^. The absorption band in the vicinity of 1650.77 cm^−1^ represents the stretching vibration of the C=O group^28^. CH_2_ and OH bonding (Fig. 2) were depicted by the absorptions around 1419.35 cm^−1^. Strong absorption at 1077.05 and 1045.23 cm^−1^ was ruled over by vibration-stretching glycosidic linkage (C–O–C)^29,30^. The absorption band at 917.452 cm^−1^ indicates the vibrations of the glycoside bond C–O–C^31^. The presence of sulfated groups and/or the presence of polysaccharides are indicated by small peaks in the fingerprint region (region less than 1500 cm^−1^)^32^. The characteristic band for α-D glucan is located at 833.098 cm^−1^. The presence of carboxyl groups in the FT-IR spectra of the polymer indicates that they may be used as an active site for the divalent cations^33^ to scavenge DPPH radicals.

### Antioxidant potential of PsPc-3

**Figure 3:**
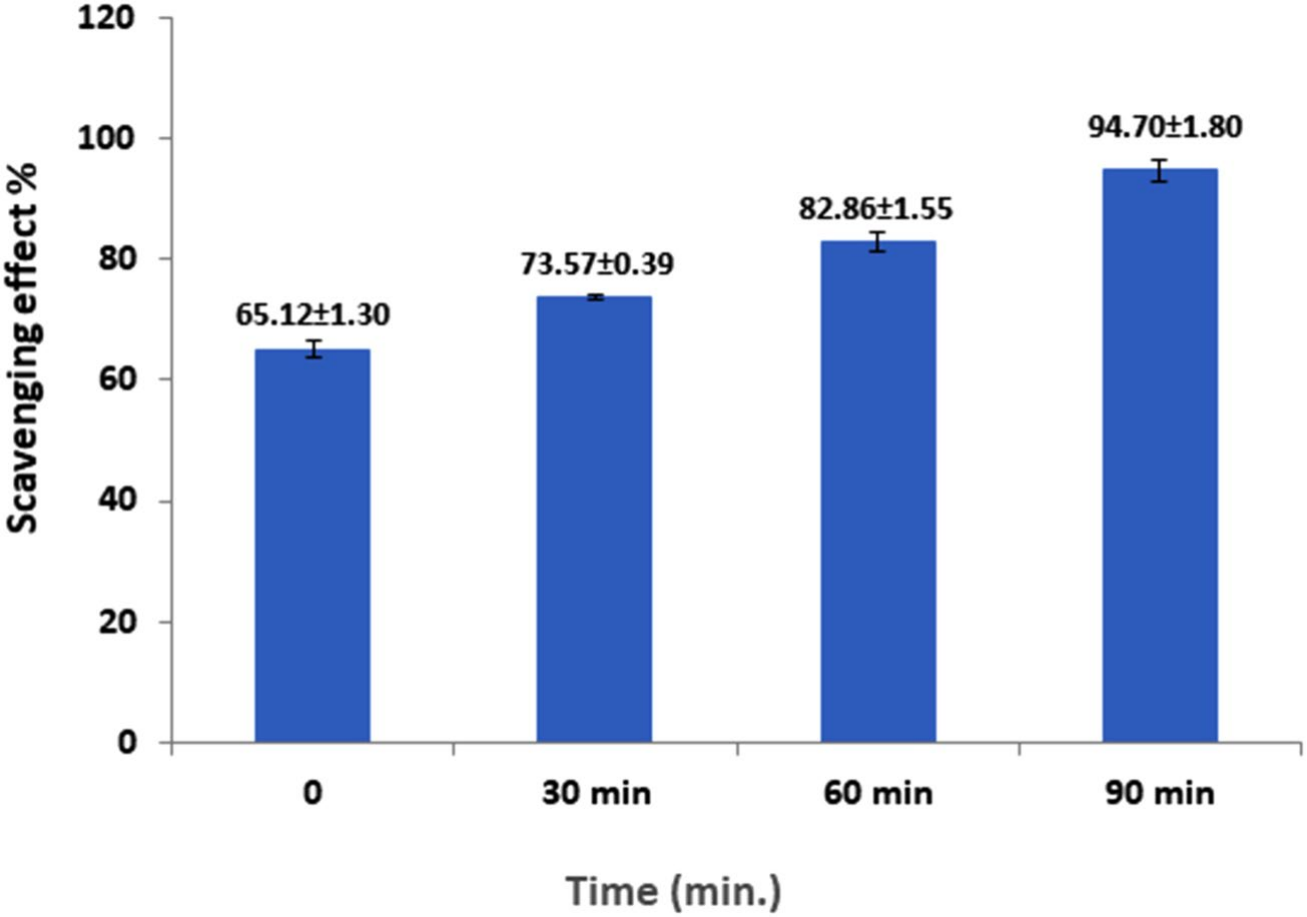
DPPH scavenging activity of the PsPc-3 from *Pleurotus columbinus.*

Owing to its simplicity and reproducibility, the DPPH free radical assay is frequently used to evaluate the free-radical scavenging capacities of diverse natural substances. Results in Fig. 3, indicated that polysaccharide's DPPH radical scavenging activity was 65.12–94.7% at 10 mg/ml concentration. There was a finding that an increase in the incubation time increased the high degree of activity. The maximum inhibition was 94.7% after 90 min, and the lowest inhibition was 65.12% at zero time. By interacting with reactive oxygen species or by reduced oxidation of metabolites, antioxidants halt the generation of oxygen free radicals. Because the size of the carbohydrate molecule has the biggest impact on the polysaccharide's capacity to scavenge free radicals, polysaccharide have better antioxidant capabilities than monosaccharides. The presence of other antioxidant components in the crude polysaccharide, such as microelements, which work in concert or interact with other substances present in the polysaccharide that has been partially purified, may be responsible for the significant action^34^.

### Body and kidney weight

**Figure 4:**
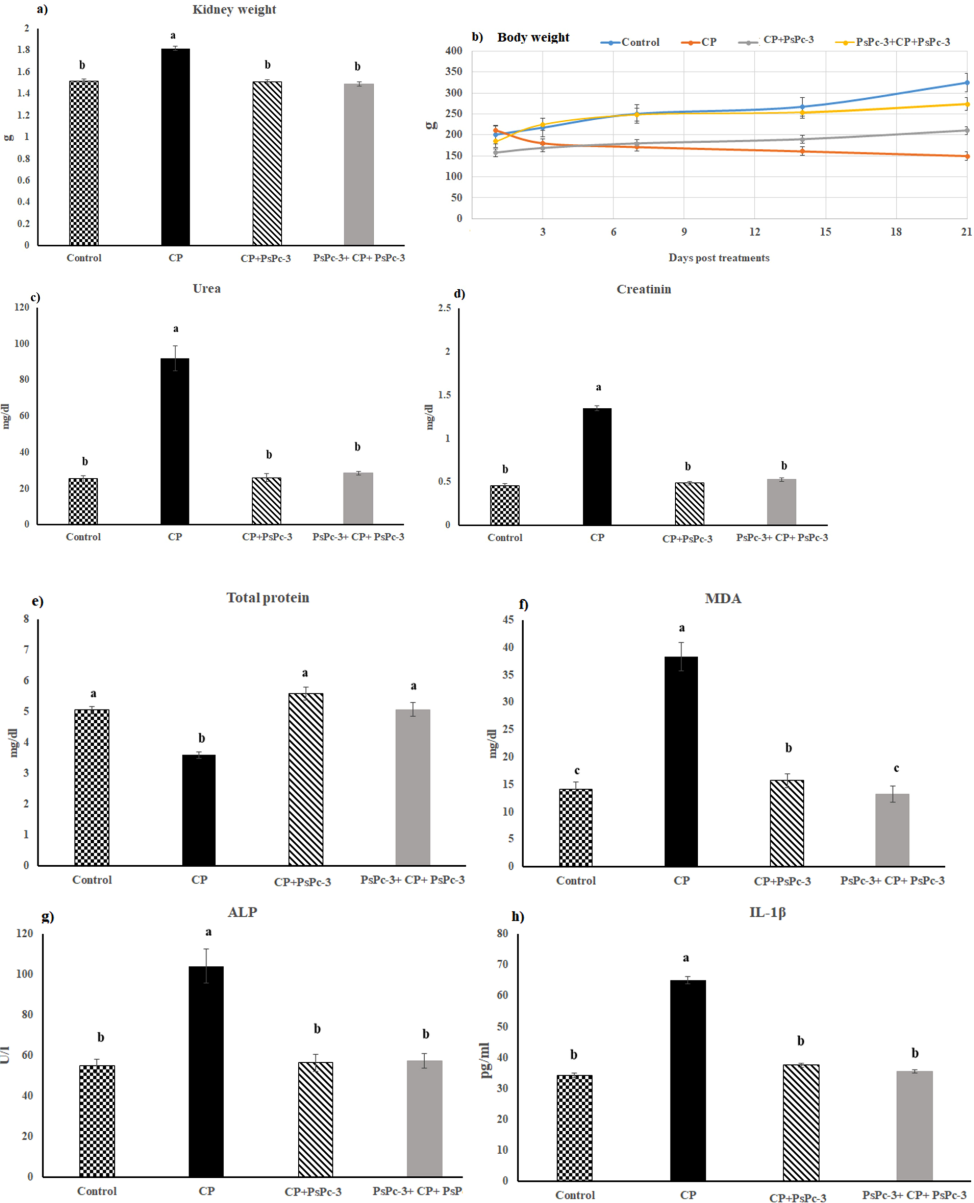

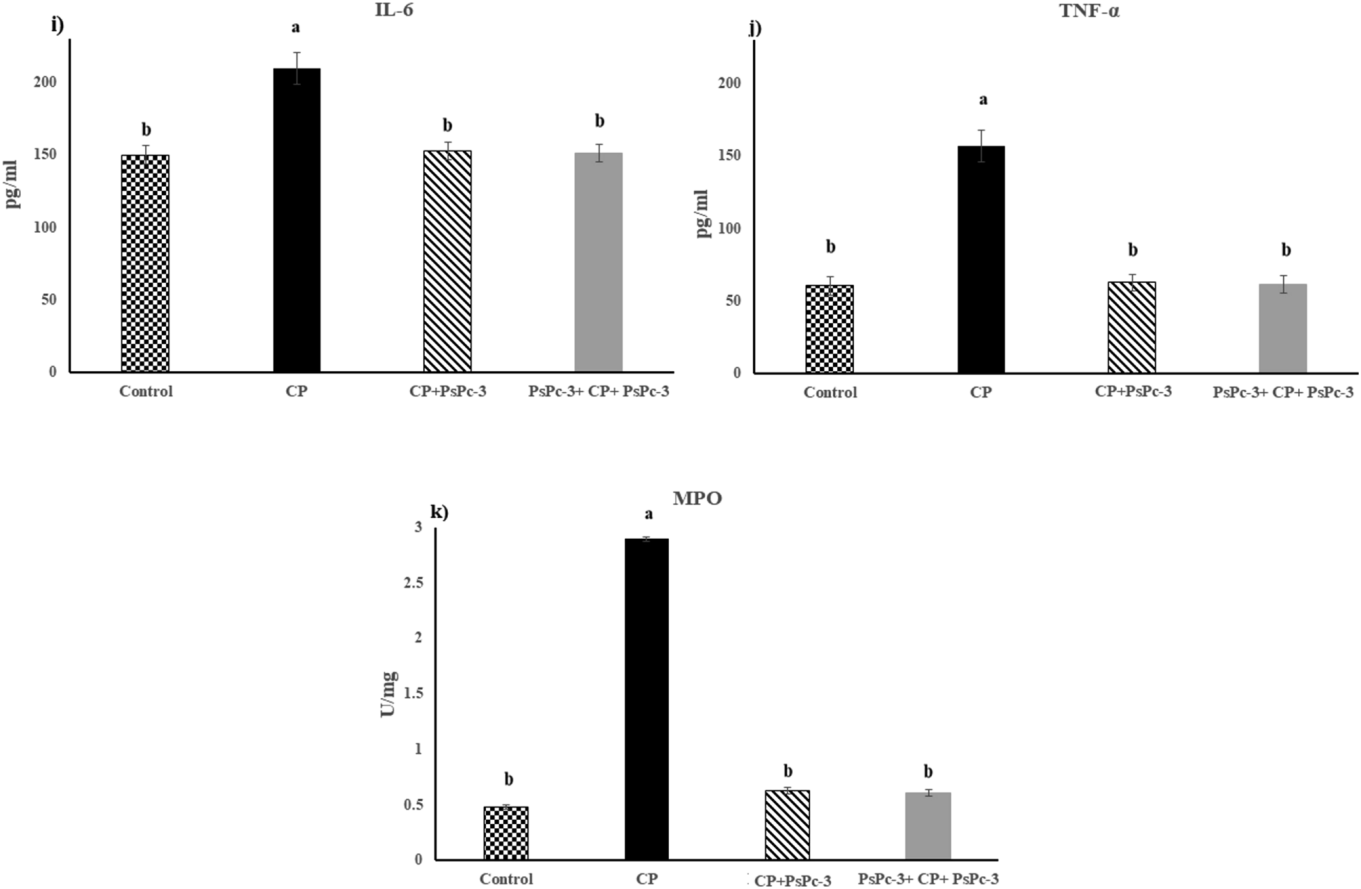
(**a**) Effect of PsPc-3 against CP on kidney weight, (**b**) Body weight, (**c**) Urea, (**d**) Creatinine, (**e**) Total protein, (**f**) MDA, (**g**) ALP, (**h**) Proinflammatory cytokines, i) MPO. The data are means ± SE (*n* = 8). One-way ANOVA followed by Tukey’s multiple comparison post hoc test; the differences were considered significant at *P* ≤ 0.05. In the kidney weight, urea, creatinine, ALP, and proinflammatory cytokines (^a^*P* < 0.05 versus Control, CP + PsPC-3, and PsPc-3 + CP + PsPc-3, ^b^*P* > 0.05 vs. Control); in total protein (^a^*P* > 0.05 vs. Control and *P* < 0.05 vs. CP only, ^b^*P* < 0.05 vs. Control, CP + PsPC-3, and PsPc-3 + CP + PsPc-3. In MDA (^c^*P* > 0.05 vs. Control, ^a^*P* < 0.05 vs. Control, ^b^*P* < 0.05 vs. CP alone treated group). Therefore, small letters (a, b, c) indicate a statistically significant difference.

The weight of the body was significantly less in the CP-treated groups, but the kidney weight increased than in the control group. Furthermore, body weight increased in both of the CP + PsPc-3- and PsPc-3 + CP + PsPc-3-treated groups, but the kidney weight significantly decreased as compared to CP- control group (Fig. 4a,b), respectively.

### Kidney functions

Cisplatin administration induced severe biochemical changes as well as oxidative imbalance in the kidney. Serum levels of creatinine and urea were assessed as markers of renal functions. Cisplatin groups showed a significant increase (*P* < 0.05) in serum creatinine F (3, 16) = 6.02 and urea levels F (3, 16) = 74.83 compared to control group. Concomitant treatment of rats with PsPc-3 significantly (*P* < 0.005) reduced the elevated amounts of urea and creatinine in the serum of cisplatin treated group to near control (Fig. 4c,d). In addition, Results revealed that total protein level was significantly F (3, 16) = 9.20 (P < 0.05) decreased in cisplatin treated rats as compared to control and treated with PsPc-3 groups (Fig. 4e).

### Lipid peroxidation

Renal malondialdehyde was assessed as an index of renal lipid peroxidation. There was a significant increase of CP on MDA at the P < 0.05 level for the three conditions F (3, 16) = 33.05. PsPc-3 treatment before and after cisplatin group significantly suppressed lipid peroxidation in comparison with cisplatin group (Fig. 4f). The activity of alkaline phosphatase was markedly increased in cisplatin-treated group when compared with control F (3, 16) = 20.50 (P < 0.05), while this activity was restored to near control values by polysaccharide administration (Fig. 4g).

#### Pro and inflammatory mediators

Figure 4h–j showed significant elevation in IL1β, IL6, and TNF-α levels in cisplatin treated rats as compared to control F (3, 16) = 35.12, 53.73, and 22.04, respectively (*P* < 0.05), and treated with PsPc-3 groups. Likewise, abuse of cisplatin led to a significant increase in myeloperoxidase F (3, 16) = 41.06, (*P* < 0.05), when compared to the control or treatment with PsPc-extract groups. In the same way, a significant increase in myeloperoxidase (MPO) occurred due to the effect of cisplatin. Interestingly, PsPc-3 administration significantly improved cisplatin-induced alterations in Myeloperoxidase (MPO), compared to the corresponding values for the untreated cisplatin group, with the return to normal control values in the Cis + PsPc-3-treated groups (Fig. 4k).

### Antioxidants

Table 1 illustrates the abnormally low levels of antioxidant enzymes (SOD, CAT, and GPx) and non-antioxidant enzyme (GSH) that are a characteristic of kidney injury caused by cisplatin. The activities of these antioxidants were markedly decreased in cisplatin treated Group when compared with control and these parameters were restored to near control values by PsPc-3 co-administration.

**Table 1:** Effect of PsPc-3 on SOD, SOD, GPx, and GSH.

Parameters	Control	CP	CP + PsPc-3	PsPc-3 + CP + PsPc-3	F
SOD (U/g)	10.27 ± 0.63^a^	4.06 ± 0.08^b^	11.14 ± 0.56^a^	13.22 ± 0.77^a^	37.42*
CAT (U/g)	21.86 ± 1.01^a^	12.57 ± 0.56^b^	20.43 ± 1.33^a^	24.57 ± 1.54^a^	16.77*
GPx (U/g)	30.57 ± 2.07^a^	16.14 ± 1.35^b^	34.29 ± 1.62^a^	29.08 ± 0.81^a^	42.71*
GSH (U/g)	34.57 ± 1.57^b^	21.71 ± 0.78^c^	36.86 ± 2.12^ab^	40.14 ± 2.15^a^	39.51*

The data are means ± SE (*n* = 8). One-way ANOVA followed by Tukey’s multiple comparison post hoc test; the differences were considered significant at *P* ≤ 0.05. Small letters (a, b, c) indicate a statistically significant difference. F (*) indicates a statistically significant difference.

In cisplatin-treated rats, there is significant changes in renin and aldosterone were noticed F (3, 16) = 94.35 and 30.01 (P < 0.05), treatment with PsPc-3 modulated these effects if compared to the non-treated group (P < 0.05) (Table 2).

**Table 2:** Effect of PsPc-3 on renin and aldosterone.

Parameters	Control	CP	CP + PsPc-3	PsPc-3 + CP + PsPc-3	F
Renin(ng/ml)	3.15 ± 0.11^b^	15.5 ± 1.08^a^	3.44 ± 0.27^b^	4.51 ± 0.48^b^	94.35*
Aldosterone (pg/ml)	489.20 ± 11.49^b^	1205.48 ± 112.01^a^	496.81 ± 50.47^b^	500.80 ± 39.62^b^	30.01*

The data are means ± SE (*n* = 8). One-way ANOVA followed by Tukey’s multiple comparison post hoc test; the differences were considered significant at *P* ≤ 0.05. Small letters (a, b) indicate a statistically significant difference. F (*) indicates a statistically significant difference. ^a^*P* < 0.05 versus control, CP + PsPc-3, and PsPc-3 + CP + PsPc-3, ^b^*P* > 0.05 versus control.

### DNA fragmentation as an index of cisplatin-induced apoptotic activity

**Figure 5:**
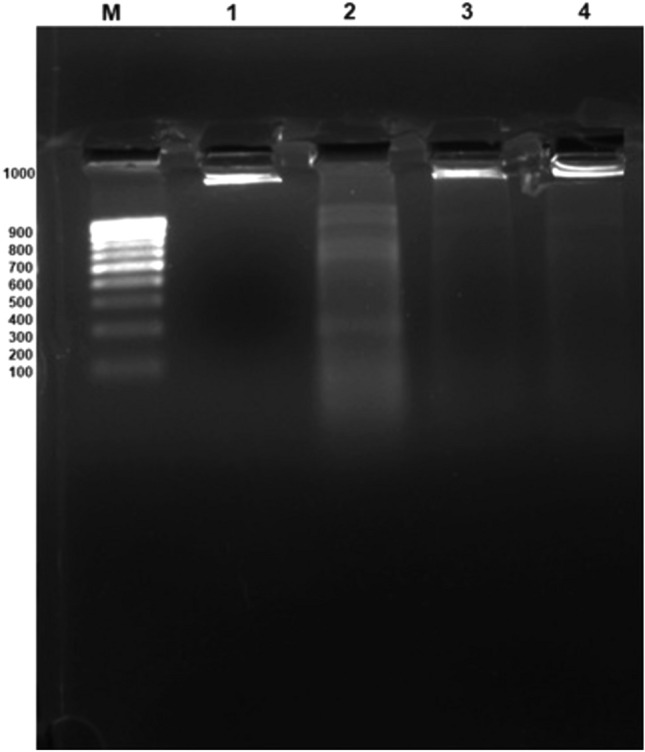
Agarose gel electrophoresis of the testicular DNA showing the activity of the PsPc-3 extract investigated in this study. Lane 1: the control group. Lane 2: CP-induced OKI in rats. Lane 3: the treatment with the PsPc-3–EtOH extract at the dose of 100 mg/kg post-3 days from the exposure to CP. Lane 4: the treatment with the PsPc-3 extract at the dose of 100 mg/kg before and after CP. M: marker (1 kb DNA ladder).

The adverse apoptotic effects of CP in animal rats were mostly observed in this work utilizing the DNA fragmentation approach (Fig. 5), where the DNA ladder represented a succession of pieces that are multiples of 180–200 bp. When animals were exposed to a single dosage of 6 mg/kg CP, the diffuse pattern of DNA fragments was clearly visible, when compared to the control. In contrast, there was a significant reduction in DNA degradation following treatments with various doses of polysaccharide extract (post, pre and post). The anti-apoptotic protection was mostly based on the kind of seaweed extract utilized, with the 400 mg/kg ethanolic extract showing the most pronounced impact on reducing DNA structural damage after 60 days of therapy.

### Histopathological findings of kidney tissue

**Figure 6:**
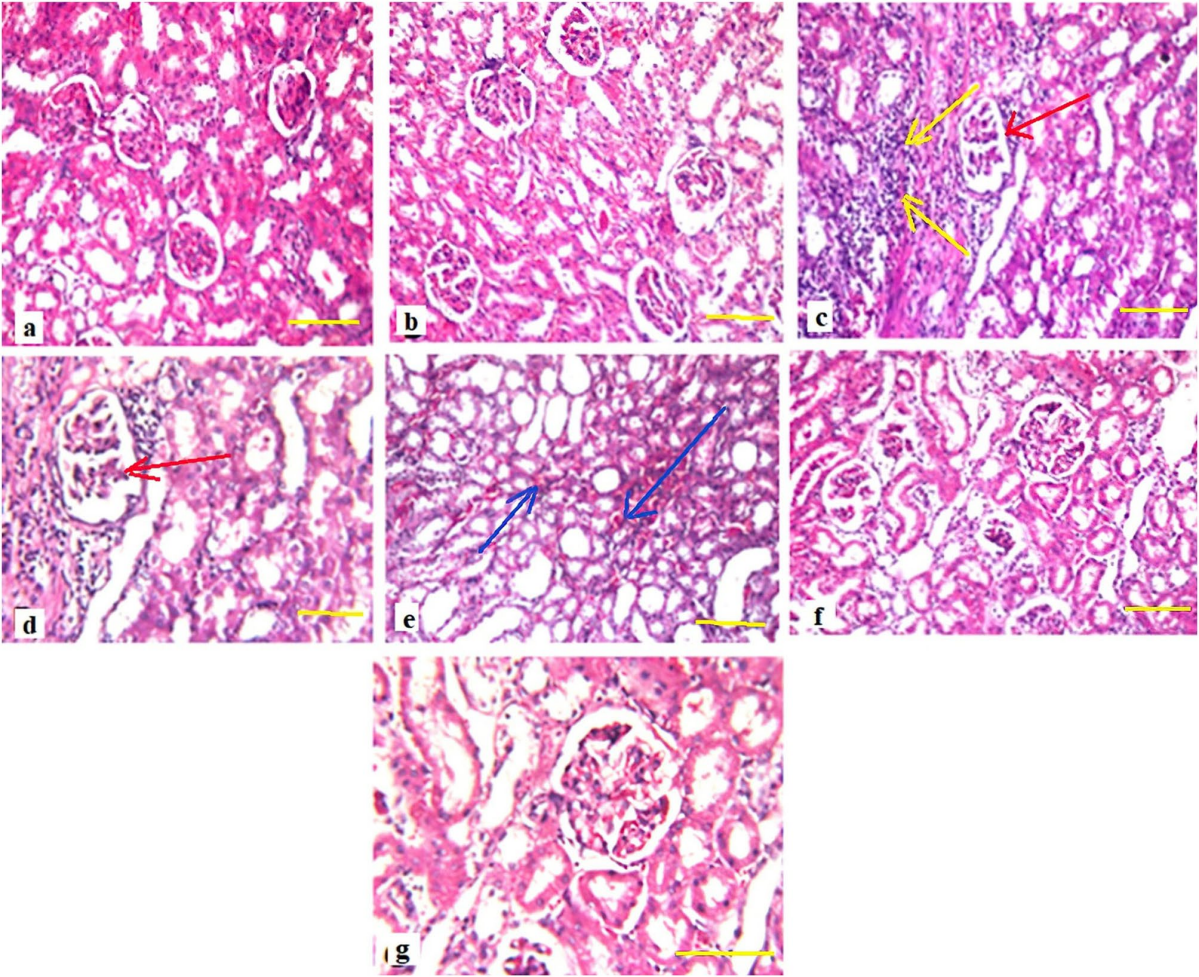
Light microscopy (LM) images showing the morphology of the normal sperms and their various defects; (**a**) normal kidney, (**b**) normal rats administered with the extract PsPc-3; (**c**,**d**) showing the degenerative change in the lining epithelium of some tubules at the cortex (red arrows) and focal inflammatory cells infiltration with fibrosis (yellow arrows) were detected in between the glomeruli and tubules at the cortex after the treatment with CP, also (**e**) the corticomedullary portion showed focal haemorrhage in between the tubules (blue arrow); (**f**,**g**) There was no histopathological alteration as recorded in the group was treated with the PsPc-3 extract at the dose of 100 mg/kg before and after CP (Giemsa stain; scale bare 50 µm).

There was no histopathological alteration and the normal histological structure of the glomeruli and tubules at the cortex were recorded in control group and group of normal rats treated with PsPc-3 (Fig. 6a,b respectively). While rats, which administered with cisplatin revealed focal inflammatory cells infiltration with fibrosis in between the glomeruli and tubules at the cortex, and degenerative change was noticed in the lining epithelium of some tubules at the (Fig. 6c,d). The corticomedullary portion showed focal hemorrhage in between the tubules (Fig. 6e) post CP group. On the contrast, in polysaccharide (before and after)-group there was no histopathological alteration as recorded in (Fig. 6f,g).

## Discussion

One of the most successful cancer treatments is the drug cisplatin, which is used to treat many solid tumors such as urinary system cancers^35^. However, one of the most significant cisplatin side effects is nephrotoxicity, and this is due to its accumulation in the kidney tissues^36^. The mechanism of nephrotoxicity-induced cisplatin is complex and not completely understood. Several cellular processes, including an aspect of increased oxidative stress and inflammation as well as programmed cell death, have been found involved^37^. Successive doses of cisplatin lead to progressive and irreversible nephrotoxicity, including the use of preventive measures^38^. In the current research, a single dose of cisplatin (6 mg/kg) revealed important declines in the body's weight and kidney, the renal CAT, SOD, GSH, and GPx antioxidant enzymes, also DNA structure, ultimately resulting in AKI. These findings are closely similar to those reported from both animal studies^39^ and research involving humans^40^, but some of these researches used different oxidative stress factors. The quest for new pathways to avoid nephrotoxicity due to cisplatin is, therefore, a promising option.

In this study, we have demonstrated that PsPc-3’s protective function is to protect the tubular kidney against apoptosis in cisplatin-induced AKI. The phytochemical discoveries have led to the discovery of active substances in plants used in traditional remedies and to rely on them in the manufacture of a large proportion of medicines of plant origin^41^. Mushrooms are naturally capable of accumulating minerals and vitamins, as well as a variety of secondary metabolites including organic acids, alkaloids, phenols, and terpenoids^42^. Mushroom polysaccharides differ from other types of polysaccharides in regards to molecular weights, chemical characteristics, degree of branching, skeleton lengths, three-dimensional orientation, and other properties^43^. The administration of PsPc-3 extract significantly modifies the dysfunction of nearby renal tubular cells by slightly restoring the indicators of renal function in the blood to their normal limits and restoring the activity of the antioxidant system. We recorded the ability of this extract to modulate kidney function, which in turn improved kidney weight and the decline of inflammatory mediators. The antioxidant role of the mushroom extract as it contains polyphenols, which are the largest, broadest, and most common group known to inhibit free radicals^44^. Thus, oxidative stress is inhibited, as reported in a study by^45^. the imbalance of antioxidants and the generation of several harmful free radicals, including the hydroxyl radical OH, superoxide anion O_2_, and hydrogen peroxide H_2_O_2_^46^. Because of kidney failure, concentrations of renal antioxidant enzymes (SOD, CAT, GSH, and GPx) were significantly reduced, as was apoptotic DNA fragmentation. Administration of a dose of PsPc-3 extract of 100 mg/kg for 21 days significantly alleviated CP-induced kidney failure in rats, as evidenced by relieving and regulating all of the morphological, enzymatic, and DNA apoptotic features stated above. Chemotherapy, including CP, is distinguished by its devious ability to generate a high quantity of ROS, which has vandal effects on the cellular DNA, generating a range of biological repercussions such as cell death and inflammation, leading to impairment of numerous organs and systems.

The reducing power, and DPPH, hydroxyl, and superoxides scavenging activities, are frequently used to examine antioxidant activity in vitro. Many studies have found that fungi polysaccharides have antioxidative effects on these free radicals, which can induce lipid peroxidation and result in a broad range of diseases^47,48^.

The analysis's findings demonstrated the polysaccharide's capacity to neutralize DPPH because the polysaccharide's antioxidant activity was shown by the IC50 value was 65.21–95.51% at 10 mg/ml. Polysaccharides are the primary biocompatible constituent of mushrooms, with antioxidant, anti-inflammatory, antitumor, immunomodulatory, and other activities such as antifungal properties. Pleurotus mushrooms are considered a safe therapeutic arbitrator to be consumed as a functional food due to their biological activities^49^.

Oxidative stress and the consequent lipid peroxidation may participate when free oxygen radicals have produced that interact with lipid membranes, leading to the formation of MDA and causing kidney damage^50^. The results of our experiment aiming to study the protective role of the PS against cisplatin's toxicity (at a level of 6 mg/kg) demonstrated its capacity for the PS (100 mg/kg) to reduce the MDA values. Polysaccharides can reduce oxidative damage by antagonizing the decrease in MDA content. Pleurotus polysaccharides have been shown in studies to reduce the body's peroxidative damage by eliminating free radicals^51^. Although the current study concluded a significant recovery effect on kidney function in the group that took the extract before and also before-after cisplatin, there are previous studies that evaluated proactive prevention with these components as more effective than the same treatments before and after cisplatin dam^52^.

## Methods

### Extraction of mushroom sample

Dry powder of mushroom sample 200 g was accurately weighed and set into a conical flask 5 l then 2 l distilled water was added for the preparation of water extracts^53^. The sample was collected for 24 h and then centrifuged at 5000 rpm for 10 min with a centrifuge (Sigma-Laborzentrifugen, 2K15). The supernatant (phenolic extract) was transferred to another flask and deposited at − 20 °C. After extraction of phenolic compounds, the remaining residue was used to extract polysaccharide by adding 2 l of distilled water and placing it for 4 h in a boiling water direction, then centrifuged by a centrifuge (Sigma Laborzentrifugen, 2K15) at 5000 rpm for 10 min^53^.

### Isolation and fractionation of PsPc-3

The supernatant was treated with three volumes of ethanol to precipitate polysaccharide. The polysaccharide was extracted from the supernatant, and TCA 5 percent was deproteinized^54^. Afterward, five volumes of cold ethanol had been used to precipitate polysaccharide. The precipitation resulted in centrifugation, re-dissolution in water (deionized), and dialysis for 3 days at a temperature of 4 °C. The residues were centrifuged for extracting the insoluble content, and then split with cold ethanol in 1, 2, 3, and 4 volumes. For further work, fractions of the powder were collected^55^.

By using glucose as normal through the phenol–H_2_SO_4_ method total sugars were calculated^56^. The m-hydroxyphenyl technique was used to measure uronic acid using glucuronic acid as the reference^57^. Sulfate was determined using a turbidimetric procedure after hydrolysis (6 N HCl, 100 °C, 5 h), using sodium sulphate as the standard^58^. High-performance liquid chromatography (HPLC) has established the monosaccharide composition of the main fraction (PsPc-3). Approximately 200 mg hydrolyzed in a sealed tube for 12 h, by using 2 ml of 3 N HCl, 100 °C. A large part of the acid was removed in the water bath at 50 °C by flash evaporation and then evaporated at the drying temperature^59^. Aminex carbohydrate HP-87C column (300 × 7.8 mm) was used for the HPLC analysis, with deionized water serving as the mobile phase and a flow rate of 0.5 ml/min/°C. Through comparison with authentic sugars, the sugar was identified.

### Molecular weight analysis

The average molecular weight (Mw) and average molecular mass (Mn) of polysaccharide were calculated using an Agilent 1100 HPLC system with a RI detector. Before injection, the polysaccharide has been dissolved in 2 ml of solvent and filtered through a 0.45 filter. The polydispersity index (PI) calculated from the Mw/Mn ratio^60,61^.

### FT-IR spectrum

Scientific Bucker 500-IR FTIR (Bucker Co., Ettlingen, Germany) spectrophotometer with a length of 4000–400 cm^−1^ was used to evaluate the PS spectrum. The purified polysaccharide was ground with KBr powder of spectroscopic consistency and then squeezed into pellets for FTIR measuring^62^.

### Antioxidant potential analysis

The method described by Brand-Williams et al.^63^ was used to test the methanolic mushroom polysaccharide's capacity to donate hydrogen atoms or electrons by bleaching the purple methanol solution of 1,1-Diphenyl-2-picrylhydrazyl (DPPH). The stable radical DPPH serves as the reagent in this spectrophotometric test. A solution of DPPH (0.1 mM) in methanol was prepared, and 1 ml of this solution was added to 1 ml of the methanolic polysaccharide of *Pleurotus columbinus* at a concentration of 10 mg/ml. The mixture was forcefully shaken and let to cool to room temperature. In a UV–VIS Spectrophotometer (Systronics, Model 119) at various times over (zero, 30, 60, and 90) min, the absorbance was measured at 517 nm. The reaction mixture's lower absorbance revealed a higher level of free radical scavenging activity. This equation was used to determine the ability to scavenge the DPPH radical:

$${\text{DPPH scavenging effect}}\;{\text{(\% )}} = \left[ {\left( {{\text{A}}0 - {\text{ A}}1/{\text{A}}0} \right) \times 100} \right]$$

where A0 is an absorbance of control reaction and A1 is the absorbance in the presence of sample of the mushroom polysaccharide. The mushroom polysaccharide's antioxidant capacity was quantified using an IC50 value and compared to the accepted standard. The amount of an extract (in mg/ml) that scavenges DPPH radicals by 50% is known as the IC50 value.

### In vivo experiment

#### Animals

In the present study, 32 adult male Sprague Dawley rats weighing 200–240 g, aged 6–7 weeks, were used. The rats were obtained from the animal facility of Cairo University's Institute of Ophthalmology. Rats were fed and given water ad libitum on a standardized laboratory healthy diet. The rats were kept at a temperature of 20 °C and subjected to a 12-h light/dark cycle.

The experimental protocol conducted in the study complies with the ethical guidelines and the principles for the care, use, and handling of experimental animals’ guidelines that following the Guide for the Care and Use of Laboratory Animals (NIH publication No. 85-23) were followed, as well as specific national laws where applicable. The protocol was revised and approved by the Ethics Research Committee of National Center for Radiation and Technology, Egyptian Atomic Energy Authority Cairo, Egypt. This research was done in compliance with the ARRIVE guidelines and regulations.

#### Experimental design

Cisplatin-induced nephrotoxicity was performed by a single intraperitoneal (i.p.)^64^ cisplatin injection (6 mg/kg in 0.9% saline).

**Figure 7:**
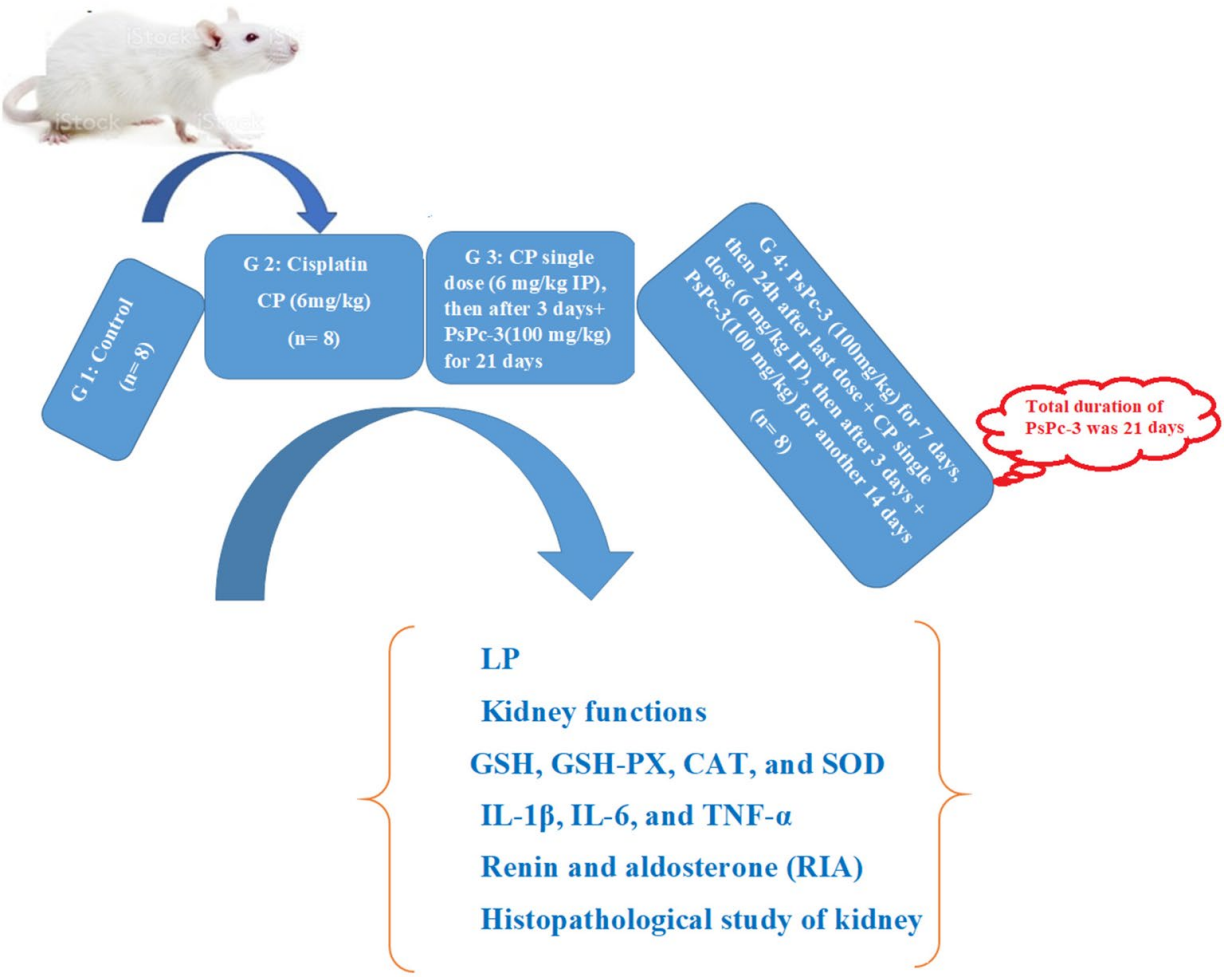
Design in vivo experiment.

Male rats were randomly selected and split into four groups, each comprising eight animals, and treated for 21 days as consequently:

- Group 1: Rats were orally treated with saline.
- Group 2: Rats were injected intraperitoneally with 6 mg/kg cisplatin (Sigma-Aldrich Co, St Louis, MO, USA) in 0.9% saline.
- Group 3: Rats were co-administered orally with PsPc-3 100 mg/kg ethanol extract^65^ (according to a preliminary experiment) post the third day of cisplatin injection^66,67^ for 21 days.
- Group 4: Rats were co-administered PsPc-3-treated 7 days before cisplatin treatment and cisplatin was administrated as in the cisplatin group, then PsPc-3 administration was continued for another 14 days post the third day by cisplatin injection (Fig. 7).

#### Blood assays and tissue sampling

Blood samples from the retro-orbital plexus were captured at end of the trial (24th day) and centrifuged to sera at 3000 rpm. They were anesthetized by using a combination of ketamine and 2 percent Rompun^®^ (100 and 20 mg/ml respectively) anesthetized. 

The kidney was rapidly removed, cleaned with physiological saline, blotted with filter paper, weighed, and homogenized in ice-cold saline (0.9 NaCl). At 10,000 rpm, the homogeneous was centrifuged at 4 °C for 10 min, and the supernatant was set up for testing.

#### Assessment of lipid peroxidation (LP)

The thiobarbituric acid reactant (TBAR) is an effective way to detect lipid oxidation^68^. This assay tests malondialdehyde (MDA), results from the lipid oxidation of polyunsaturated fatty acids. The MDA responds to the formation of a pink chromogen (TBARS), which is estimated at 532–535 nm.

#### Determination of kidney functions

Total protein assessment was performed using the technique of Doumas^69^, albumin was also measured in rat serum^70^. Serum urea and creatinine analysis were also done using the technique of^71^. Biovision (Avenue, USA) kits were used to test serum alkaline phosphatase (ALP).

#### Determination of antioxidants

The extent of GSH-Px was determined using a spectrophotometric kit (Randox Laboratories, Crumlin, UK) using a technique developed by^72^ in which GSH-Px catalyzed GSH oxidation with GSH reductase, cumene hydroperoxide, and NADPH. The oxidised GSH was quickly decreased, and the amounts of GSH-Px (U/g protein) in the sample were lowered by the concurrent oxidation of NADPH to NADP+, resulting in reduced absorbance at 340 nm.

Glutathione reduced was consistent with the method reported by^73^ using the kit obtained from Biodiagnostic, Egypt**.** The spectrometric approach was applied to determine SOD activity using a commercial Ransod kit (Randox Laboratories, United Kingdom). Xanthine and xanthine oxidase are the tool for the production of superoxide radicals, which react in the red formazan dye with 2-(4-diodenyl)-3-(4-nitrophenol)-5-phenyltetrazolium chloride. The findings have been expressed as U/g protein.

Depending on the method of^74^ estimated CAT activity. Spectrophotometrically tracked at 240 nm (Shimadzu UV 1601, Japan), the decomposition of the substrate H_2_O_2_ has been observed. A change in absorption was calculated by CAT activity and finally expressed by U/g tissue.

#### Pro and inflammatory biomarker evaluation

TNF- α, IL1β, and IL-6 levels was measured using commercially available kits (ABCAM, Kendall Square, Suite B2304, Cambridge, USA, Cat.# ab 236712, 255730 and 100772, respectively) by an enzyme-linked immunosorbent assay (ELISA). TNF-α, IL-1β, and IL-6 levels were expressed in pg/ml. While the Granell et al.^75^ method was used in determining myeloperoxidase (MPO) (CUSABIO Technology LLCCSB-E08722r).

#### Renin and aldosterone

A competitive radiation immunoassay (RIA) is used in the present process^76^. During incubation, for the particular sites of the antiserum coated on the tubes, the sample/calibrator aldosterone and Renin competes with the aldosterone labeled with Iodine 125 (tracer). Separation consists of the application of antimicrobial coated tubes in which anti-aldosterone is attached to the tube walls. The radioactivity is measured in the tubes in a gamma counter after suction. The binding degree is inversely proportional to the concentration of the sample/calibrator’s hormone.

#### DNA fragmentation by agarose gel electrophoresis (qualitative analysis)

The extent of DNA fragmentation in the kidney tissue was determined by the method described by Bohlinge et al.^77^.

Briefly, kidneys were removed and utilized for DNA isolation by PBS-E homogenization (50 mM sodium phosphate buffer containing 0.9 percent saline and 20 mM EDTA, pH 8)^77^. They were suspended in 2 ml of PBS-E with 0.5 mg/ml collagenase. The Ultra Turrax homogenizer (IKA T 25-German) was used, and ice testing specimens were kept before and after homogenization. A stirring followed by a pronase E addition (1 mg ml) was incubated for 1 h at 37 °C, followed by a 15-min incubation at 37 °C. The suspension was then centrifuged for 5 min at 1000×*g*. A pellet was dispersed and infused with 2 ml lysis buffer comprising 50 mM Tris–HCl, pH 8, 20 mM EDTA, 10 m M NaCl, and 1% w/v SDS and centrifuged for 15 min at 14,000×*g* with phenol–chloroform extraction. DNA was dissolved by moderate shaking at 65 °C in 10 mM Tris–HCl, pH 8, adding 1 mM EDTA. The standard procedure for this technique^78^.

### Histological analysis

Kidney samples of rats were fixed with the 10% formal saline, which were then dehydrated at graded alcohol levels. Further cleared with xylene solution three times and was then trapped in paraffin wax. The microtome was then used on a slide to cut 4–5 mm of para-n waxed tissue and was stained with hematoxylin (H) and eosin (E). Using a light microscope (Olympus CH; Olympus, Tokyo, Japan), the slides were then further examined^79^.

### Statistical analysis

The results were expressed as mean ± standard error (S.E.) for eight animals in each group. Differences between groups were assessed by one-way analysis of variance (ANOVA) when differences were significant. Data were statistically analyzed using SPSS version 23 (SPSS, Cary, NC, USA). The variations Duncan's test used for multiple comparisons between groups.

### Statement of ethics

The guidelines for the ethical use and maintenance of laboratory animals issued by the Guide for the Care and Use of Laboratory Animals (NIH publication No. 85-23), and authorized by the Nuclear Research Center, Egyptian Atomic Energy Authority, Cairo, Egypt, were complied with in all procedures used in caring for rats and taking blood and tissue samples for this experiment.

## Conclusions

The study of indicators associated with nephrotoxicity is broad and multifactorial. Many factors contribute to the development of these diseases, which are the principal reasons for renal failure. The majorities of natural antioxidants are the source of medicinal plants and are still applied in the medical field. Note that they are antioxidants that significantly prevent several diseases, including kidney failure, and studies have been directed towards developing new drugs that depend on antioxidants of natural origin and dependence on them in the medical field. The current research showed that treatments with PsPc-3 extract both post- and pre-post-cisplatin improved renal function in rats as demonstrated by modulating levels of antioxidant kidney enzymes (SOD, CAT, GSH, GPx), and death. DNA apoptosis and markers for inflammation. These results were likely attributed to the synergistic therapeutic effects of the natural antioxidants found in the mushrooms, as they regulated the various physiological and morphological aspects of the oxidative stress and damage that occurred in the kidneys. Therefore, it has a promising protective ability against chemotherapy and can be suggested to be a potential therapeutic option for protecting the kidneys induced by cisplatin in rats.

## Data Availability

Data is contained within the article.

## Abbreviations

CP: Cisplatin
ORI: Oxidative renal injury
Mn: Molecular mass
Mw: Molecular weight
MDA: Malondialdehyde
ALP: Alkaline phosphatase
CAT: Catalase
IL-6: Interleukin-6
IL1β: Interleukin 1-beta
TNF- α: Tumour necrosis factor-alpha
PsPc-3: Polysaccharide isolated from *P. columbinus*
ARF: Acute renal failure
HepG2: Hepatoma cell
TCA: Trichloroacetic acid
H_2_SO_4_: Hydrochloric acid
PI: Polydispersity index
IR: Infrared
FTIR: Fourier transform infrared
DPPH: 1,1-Diphenyl-2-picrylhydrazyl
NaCl: Sodium chloride
GSH-Px: Glutathione peroxidase
GSH: Glutathione
NADPH: Nicotinamide adenine dinucleotide phosphate
SOD: Superoxide dismutase
RIA: Radiation immunoassay
EDTA: Ethylenediaminetetraacetic acid
HCl: Hydrochloric acid
H: Hematoxylin
E: Eosin
EPS: Exopolysaccharide
AKI: Acute kidney injury
